# The Arabic Control Attitudes Scale-Revised: Method Effect (Negatively Worded Items) and Measurement Invariance as Threats to Its Construct Validity Are Remedied in a 5-Item Version With Improved Performance

**DOI:** 10.1097/JCN.0000000000001294

**Published:** 2026-01-28

**Authors:** Amira Mohammed Ali, Saeed A. Al-Dossary, Maryam Alharrasi, Carlos Laranjeira, Ahmad Ayed, Heba Emad El-Gazar, Mohamed Ali Zoromba, Amal Diab Ghanem Atalla, Rasmieh Alamer, Khalood Al-Abri, Maha Subih, Annamaria Pakai

**Affiliations:** 1 Dr. Amira Mohammed Ali, Associate Professor, Department of Psychiatric Nursing and Mental Health, Faculty of Nursing, Alexandria University, Smouha, Egypt.; 2 Prof. Saeed A. Al-Dossary, Professor, Department of Psychology, College of Education, University of Ha’il, Saudi Arabia.; 3 Dr. Maryam Alharrasi, Associate Professor, College of Nursing, Sultan Qaboos University, Muscat, Oman.; 4 Prof. Carlos Laranjeira, Professor, School of Health Sciences, Polytechnic University of Leiria, Portugal; Centre for Innovative Care and Health Technology (ciTechCare), Polytechnic University of Leiria, Portugal; and Comprehensive Health Research Centre (CHRC), University of Évora, Portugal.; 5 Dr. Ahmad Ayed, Associate Professor, Faculty of Nursing, Arab American University, Jenin, Palestine.; 6 Dr. Heba Emad El-Gazar, Associate Professor, Department of Nursing Administration, Faculty of Nursing, Port Said University, Egypt.; 7 Dr. Mohamed Ali Zoromba, Associate Professor, Department of Nursing, College of Nursing, Prince Sattam Bin Abdulaziz University, Al-Kharj, Saudi Arabia; and Psychiatric and Mental Health Nursing Department, Mansoura University, Egypt.; 8 Dr. Amal Diab Ghanem Atalla, Assistant Professor, Nursing Administration Department, Faculty of Nursing, Alexandria University, Egypt.; 9 Rasmieh Alamer, Faculty of Nursing, Yarmouk University, Irbid, Jordan.; 10 Dr. Khalood Al Abri, Associate Professor, Department of Community and Mental Health, College of Nursing, Sultan Qaboos University, Muscat, Oman.; 11 Dr. Maha Subih, Associate Professor, Faculty of Nursing, Al-Zaytoonah University of Jordan, Amman, Jordan.; 12 Dr. Annamaria Pakai, Associate Professor, Institute of Nursing Sciences, Basic Health Sciences and Health Visiting, Faculty of Health Sciences, University of Pécs, Hungary.

**Keywords:** Arabic Patient Health Questionnaire-2, Arabic Control Attitudes Scale-Revised, heart failure, known-group validity, measurement invariance

## Abstract

**Background:**

The Control Attitudes Scale-Revised (CAS-R) is widely used to explore cardiac patients’ beliefs about their ability to manage illness. The CAS-R’s construct validity may be questionable in different cultural contexts. Conclusions/applications based on inaccurate construct validity can be misleading and incorrect.

**Objective:**

In this study, we aimed to evaluate the psychometric properties of the Arabic version of the CAS-R.

**Methods:**

Within a cross-sectional design involving 180 Omani patients with heart failure (mean age = 70.3 ± 9.8 years, 51.7% females), exploratory/confirmatory factor analysis (CFA) and multigroup CFA were used to evaluate the construct validity and measurement invariance of the CAS-R across gender and marital groups.

**Results:**

In exploratory factor analysis, 2 factors with eigenvalues >1 explained 37.9% of the variance. Despite the poor fit of the unidimensional CAS-R, CFA revealed an excellent fit of a 2-factor structure. Negative (5 and 8) and cross-loading items (1) contributed to scale variance at the configural level. Eliminating negative items and item 6 improved model fit, reliability (Cronbach’s *α* = 0.66 vs. 0.56), and invariance at all levels. In support of its convergent and criterion validity, the CAS-R 5 correlated with the CAS-R and depression (*r* = 0.953, −0.268; *P* values <.01).

**Conclusions:**

Negative items comprised a minor weak factor (helplessness) that was not stable across groups. Eliminating items 5, 6, and 8 resulted in a clean invariant short form (CAS-R 5) with superior properties that may implicate nursing decisions and interventions concerning perceived control.

**What’s New and Important**

- The Arabic Control Attitudes Scale-Revised is not unidimensional.
- Negative items (5 and 8) comprised a minor weak factor of helplessness, which was not stable across groups.
- Eliminating negative items and item 6 resulted in a pure invariant unidimensional 5-item short form, which can be easily integrated into routine care for directing nursing interventions that target self-care behaviors and patient outcomes.

By 2030, more than 8 million people are estimated to be affected by heart failure.^1^ People with that condition are overwhelmed with fears related to uncertainty that surrounds their illness (eg, their ability to meet their own self-care demands, difficulty of recognizing symptoms, fears of death). Drastic lifestyle changes may ensue as a result, such as withdrawing from social and leisure activities, resulting in increased feelings of powerlessness and loss of control.^1,2^

Individuals’ beliefs that they possess resources necessary to cope positively with negative events in their life reduce experiences of an aversive nature and is associated with lower levels of psychological distress as well as more positive health outcomes.^3,4^ Indeed, high levels of perceived control among patients with heart failure are strongly associated with health-related quality of life while low levels are associated with increased indicators of psychopathology (depression, anxiety, and stress).^2,3^ While thought to be a personality trait, perceptions of control can be manipulated. Accordingly, enhancing perceived control is a target of nursing interventions in cardiac patient care since perceived control acts as an indicator of patients’ ability to cope and thrive with their chronic illness and related demands for self-management.^1^

The Control Attitudes Scale-Revised (CAS-R) has been designed to capture cardiac patients’ beliefs about their own ability to manage their heart disease. It comprises 8 items that were obtained from the original Control Attitudes Scale (2 items only [items 7 and 8]) and the Cardiac Attitudes Index (6 items).^4^ The CAS-R has a superior performance in predicting outcomes pertaining to current health problems relative to measures of locus of control. The latter are conceptually related but apply to life in general rather than to specific health problems.^5^ Because the CAS-R is brief and can be quickly administered in various cardiac patient groups, including those with advanced conditions, it can be easily incorporated into clinical practice, especially as perceived control of heart failure may quickly reflect patients’ psychosocial adaptation, symptom status both of heart failure and comorbid mental conditions (eg, depression), self-care behaviors, and health outcomes.^6–8^ As such, the CAS-R may be a valuable asset that would aid in developing effective nursing interventions that target adherence to heart failure treatment regimens as well as support systems that would improve patients’ health and quality of life,^6^ including those with a cardiac device.^1^ Given the importance of this construct, a family version of the scale has been developed based on the same design of the patient version.^9^

Initial testing of the CAS-R was conducted through principal component analysis in 3 groups, and it revealed 2 factors with eigenvalues >1. However, the developers preferred a 1-factor structure because the eigenvalue of the second factor was slightly bigger than 1 (1.04, 1.05, and 1.06), and the elbow of the scree plot was noticed at the first factor.^4^ Psychometric investigations of the CAS-R are limited relative to other measures used in this population, and some investigations are primitive in that they do not attempt to address the structure of the scale, but rather are focused on scale translation, face and content validity,^7^ as well as reliability and criterion validity.^10^ Other subsequent investigations used exploratory methods similar to the original study,^11–13^ and they favored the single-factor structure (eg, Portuguese^11^ and Chinese^12^ versions) while otherstudies, such as the Korean version, favored the 2-factor structure, which explained 63.23% of the total variance.^13^ On the other hand, investigations involving robust techniques such as item-response theory and confirmatory factor analysis (CFA) are scarce. Nevertheless, they indicate failure of some items of the CAS-R to contribute to the underlying latent trait or identify high performers.^1,3^ Moreover, we could not locate any study examining the measurement invariance of the CAS-R since this type of testing ensures measurement stability across groups, that is, mean differences between groups are real and not attributed to measurement bias ensuing from their specific characteristics.^14,15^

Although a previous group evaluated the psychometrics of the Arabic CAS-R, testing was limited to examining its reliability, item total correlations, and correlations with anxiety, with a lack of evidence on its construct validity,^10^ restricting the utility of this construct in both research and clinical settings among the more than 22 Arab countries that harbor around 500 million people. The literature confirms that overall perceived validity is highly dependent on construct validity, with important implications particularly in health research and practice as well as in other disciplines (social sciences, psychology, psychometrics, and language studies).^5,16,17^ Accordingly, in this study, we aimed to fill a gap in the literature by expanding the evaluation of the construct validity of the CAS-R through exploratory and CFA in an Arab sample. We also aimed to evaluate the measurement stability of the scale across men and women, as well as married and unmarried patients with heart failure. Convergent validity has been emphasized as a component of construct validity by evaluating the associations of the CAS-R with its items, subscales, and shortened form. The criterion validity of the measure was extended through evaluating its association with depression, which is frequently reported as a correlate of perceived control in this patient population.^6,8^

## Methods

### Design, Sample, and Procedure

In this cross-sectional study we recruited a sample of 180 patients with heart failure from governmental Omani outpatient clinics and hospitals. A sample of 148 participants was determined using G*Power software (version 3.1.9.7) (Heinrich Heine University Düsseldorf, Düsseldorf, North Rhine-Westphalia, Germany) to account for a power of 0.95 (1 − *β* error probability), significance level of .01, and an effect size of *f*² = 0.15. Taking into consideration the likelihood of having 20% missing data (due to withdrawal or refusing to respond to some questions), we extended data collection to 180 participants.

Data were collected on-site through face-to-face interviews or on the phone, depending on the participants’ preferences. Inclusion in the study was limited to those diagnosed with heart failure, 18 years or older, fluent in Arabic, and willing to take part in the study. Based on a check of the medical record, patients excluded were those who had a history of cancer, Alzheimer’s disease, and chronic mental disorders (eg, schizophrenia and major depressive disorders). Patients presenting problems with hearing and communication were also excluded. Interviews were conducted by trained and certified interviewers. One of the investigators (MA) held a weekly meeting with the interviewers to resolve any issues that erupted during data collection.

The study was conducted according to the Declaration of Helsinki. All respondents were offered information on the aim of the study and data collection procedure, as well as their rights to voluntarily participate and withdraw from the study at any time. They were informed that their data were accessible only to the researchers and that data would be used solely for research purposes. The day of the data collection interview was set by patients who consented to take part in the study around a week before actual data collection. The study has been approved by the Ethics Committee of Sultan Qaboos University (CON/EA/43/2019) and the Omani Ministry of Health of Research Committee (MOH/CSR/20/11605). Respondents with high depression scores were referred to a psychiatric examination.

### Measures

The test battery administered to the subjects comprised questions inquiring about participants’ age, gender, and marital status, in addition to 2 measures.

The CAS-R is an 8-item questionnaire developed to assess cardiac patients’ perceived control of their condition, for example, “I can do a lot of things myself to cope with my heart condition, I feel lots of control, I have considerable ability to control my symptoms.” Items 5 “No matter what I do, or how hard I try, I just can’t seem to get relief from my symptoms” and 8 “Regarding my heart problems, I feel helpless” are reverse-coded. Items are rated on a 5-point response format (strongly disagree = 1 to strongly agree = 5), with total scale scores ranging from 8 to 40. Lower scores depict lower levels of perceived control and vice versa.^4^ The reliability of the CAS-R in the present sample is fair (Cronbach’s *α* = 0.56).

The validated Arabic version of the Patient Health Questionnaire-2 (PHQ-2) is an ultra-brief form of the PHQ-9, which evaluates depressive symptoms over the course of the last 2 weeks. It comprises 2 items, “losing interest” and “feeling down, depressed, or hopeless,” which are rated on a 4-point response format (not at all = 0 to almost every day = 3), with total scale scores ranging from 0 to 6. Higher scores depict higher depressive symptomatology and vice versa.^18^ The reliability of the PHQ-2 in the present sample is fair (Cronbach’s *α* = 0.51).

The CAS-R was translated from English into Arabic, with backward translation from Arabic into English according to standard procedures.^19^ Because of the high degree of agreement between the translators, minimal modifications were made.

### Statistical Analysis

Mean and standard deviation were computed to report the descriptive statistics of the continuous variables (age, CAS-R, and its subscales/shortened forms); median (Q1–Q2) was used to report the descriptive statistics of the PHQ-2 because it expressed a non-normal distribution, while frequency and percentage were used to report the rest of the sociodemographic variables (gender and marital status).

The structure of CAS-R was examined in 2 stages. In the first stage, exploratory factor analysis (EFA) was used to evaluate the scale structure based on the responses of the participants, apart from constraints determined by the theoretical model.^14^ The method of extraction used was maximum-likelihood with Oblimin rotation, Kaiser–Meyer–Olkin measure of sampling adequacy, and Bartlett’s test of sphericity. All items were included in the initial testing, while 2 or 3 items were removed from subsequent testing according to modifications informed by CFA and multigroup CFA. The aim of repeating the test was to check for changes in variance explained and the scree plot.

In the second stage, 5 structural models of the CAS-R were assessed using CFA: (1) a unidimensional model comprising the whole set of items, (2) a 2-factor structure of the whole scale that replicates the EFA model with slight modifications (cross-loadings) based on modification indices, (3) a unidimensional 6-item structure (items 5 and 8 are removed based on cross-loadings and correlated error terms from the first 2 models), (4) a unidimensional 5-item structure reported in Taiwan (items 5, 7, and 8 are removed),^3,5^ and (5) another unidimensional 5-item structure (items 5, 6, and 8 are removed following measurement invariance analysis of the second, third, and fourth models). Model fit of the CAS-R structures was defined as acceptable or good based on the critical thresholds of >0.90 and >0.95 of both Comparative Fit Index (CFI) and Tucker–Lewis Index, along with critical thresholds of <0.08 and <0.06 of both root mean square error of approximation and standardized root mean residual.^20,21^

The stability of the best-fitting structures of the CAS-R was determined through multigroup CFA by running 4 nested models that impose increasingly restrictive constraints on factor loadings (*λ*s), intercepts (*τ*s), and residuals (*δ*s) to evaluate significant changes in model fit at the configural, metric, scalar, and strict levels across groups of gender and marital status.^22,23^ Given that *χ*^2^ test is sensitive to sample size, significant changes in model fit were defined based on critical thresholds of 3 stable (ie, independent of sample size) absolute fit indices: ΔCFI and ΔTucker–Lewis Index <0.020 and Δroot mean square error of approximation <0.015.^24^

The internal consistency of the CAS-R and its subscales/shortened forms was evaluated using Cronbach’s *α*, while item total correlations were used to assess the convergent validity of the scale at the item level.^25^ The global convergent validity of the shortened versions of the CAS-R was determined through their correlation with the whole scale.

The criterion validity of all forms of the CAS-R was evaluated through their correlation with the PHQ-2. To evaluate the known-group validity of the CAS-R, a categorical form of the PHQ-2 was constructed based on its optimal cutoff to divide the subjects into 2 groups: nondepressed (PHQ-2 score <2.5) and depressed (PHQ-2 score >2.5).^18^ Then, differences in CAS-R and its shortened forms were examined between the 2 groups through an independent sample *t* test. Because group sizes were largely different, we reported a Welch *t* test of equal variance not assumed, that is, with adjusted degrees of freedom (*df*). Statistical analyses were performed using IBM SPSS Statistics, version 25 (Armonk, New York), and IBM AMOS, version 24. Statistical significance was determined based on 2-tailed tests with a threshold of *P* < .05.

## Results

The mean age of the participants was 70.3 ± 9.8 (range = 25–90) years. The sample comprised almost equal numbers of females and males (n = 93 [51.7%] vs. n = 87 [48.3%]), and most of the subjects were married (n = 122, 67.8%). Their median (Q1–Q2) depression score was 2 (1–3). According to the cutoff score of the PHQ-2, 31.1% (n = 56) of the participants had depression and should be referred for a clinical examination to confirm the diagnosis. The descriptive statistics of the CAS-R and its items are presented in Table 1.

**TABLE 1:** Item Analysis, Internal Consistency, Convergent Validity, and Descriptive Statistics of the Control Attitudes Scale-Revised Among Omani Patients With Heart Failure (N = 180)

Items	Mean (SD)	Skewness (SE)	Kurtosis (SE)	CAS-R		Control (CAS-R 6)		CAS-R 5 Taiwan		CAS-R 5	
				ITC	*α* If Item Deleted	ITC	*α* If Item Deleted	ITC	*α* If Item Deleted	ITC	*α* If Item Deleted
1	2.92 (0.97)	−0.49 (0.18)	−0.78 (0.36)	0.54	0.38	0.60	0.57	0.59	0.49	0.60	0.51
2	2.77 (1.02)	−0.32 (0.18)	−1.03 (0.36)	0.44	0.46	0.57	0.60	0.56	0.53	0.57	0.55
3	1.88 (0.32)	−2.41 (0.18)	3.84 (0.36)	0.47	0.50	0.45	0.65	0.42	0.62	0.43	0.64
4	1.86 (0.35)	−2.11 (0.18)	2.46 (0.36)	0.48	0.49	0.47	0.64	0.46	0.61	0.45	0.63
5	1.92 (0.27)	−3.18 (0.18)	8.20 (0.36)	0.03	0.57	—	—	—	—	—	—
6	1.94 (0.23)	−3.91 (0.18).	13.46 (0.36)	0.46	0.52	0.46	0.66	0.43	0.64	—	—
7	1.92 (0.28)	−3.04 (0.18).	7.33 (0.36)	0.49	0.50	0.47	0.65	—	—	0.44	0.64
8	1.76 (0.43)	−1.20 (0.18)	−0.57 (0.36)	−0.27	0.65	—	—	—	—	—	—
Scale mean (SD)				16.97 (5.06)		13.29 (2.31)		11.37 (2.17)		11.34 (4.84)	
Cronbach’s *α*				0.56		0.68		0.65		0.66	

Helplessness: ITC = 0.22, mean (SD) = 3.68 (0.56), Cronbach’s *α* = 0.33.

Abbreviations: CAS-R, Control Attitudes Scale-Revised; SD, standard deviation; SE, standard error; ITC, item total correlation.

**FIGURE 1.**
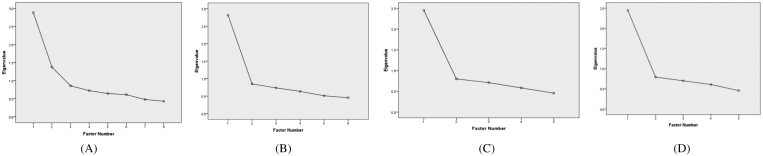
Factor structure resulting from exploratory factor analysis of the Control Attitudes Scale-Revised to classify Omani patients with heart failure (N = 180): (A) model 1 (all 8 items), (B) model 3 (item 5 and 8 removed), (C) model 4 (items 5, 7, and 8 removed), and (D) model 5 (item 5, 6, and 8 removed).

The sample size was suitable for EFA analysis as noted by Kaiser–Meyer–Olkin (0.78) and Bartlett test (*χ*^2^ = 284.48, *df* = 28, *P* < .001). Two factors with eigenvalues >1 (2.89 and 1.38) contributed to 28.9% and 9.0% of the total variance in the CAS-R. As per the scree plot (Figure 1A), the elbow shape was more distinct at the third factor rather than the second factor. All items loaded on the first factor, which may be called control, while only negatively worded items (5 and 8) loaded on the second factor, which may be labeled as helplessness/inability to control.

In repeated EFA, the sample size was suitable for EFA when 2 (5 and 8) and 3 (5, 7, and 8)/(5, 6, and 8) items were removed as noted by Kaiser–Meyer–Olkin (0.81, 0.77, and 0.77) and Bartlett test (*χ*^2^ = 238.10, 175.59, and 173.17; *df* = 15, 10, and 10; *P* < .001). One factor with eigenvalues >1 (2.82, 2.45, and 2.45) contributed to 46.94%, 49.03%, and 48.93% of the total variance in the 3 models. All items loaded significantly on this single factor (all *λ*s > 0.5) while the elbow shape of the scree plots was very distinct at the first factor (Figure 1B–D).

**FIGURE 2.**
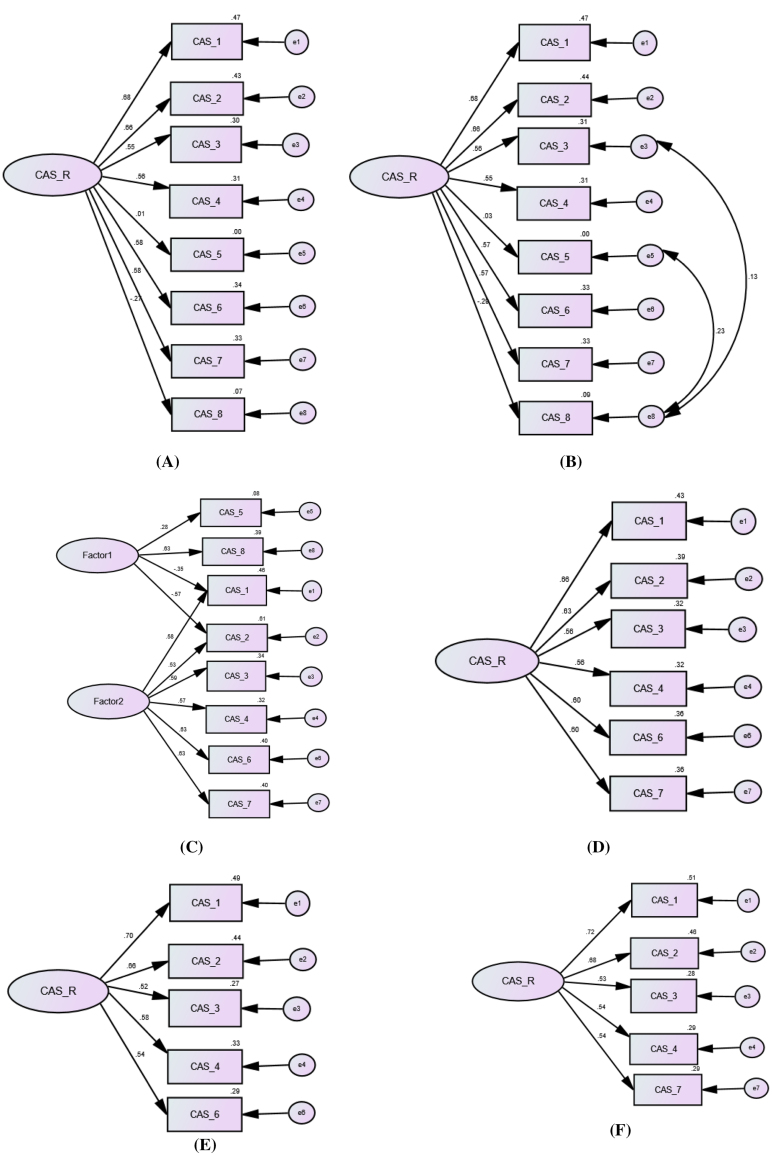
Factor structures resulting from confirmatory factor analysis of the Control Attitudes Scale-Revised to classify Omani patients with heart failure (N = 180): (A) model 1 (a crude model comprising all 8 items), (B) model 1 with error correlations, (C) model 2 (2 modified factors based on exploratory factor analysis), (D) model 3 (item 5 and 8 removed), (E) model 4 (items 5, 6, and 8 removed), and (F) model 5 (item 5, 6, and 8 removed).

CFA indicates poor fit of the unidimensional CAS-R (Table 2), even when 2 pairs of error terms were correlated (Figure 2B). While replicating the EFA model to produce 2 factors, modification indices suggested cross-loading items 1 and 2 on the helplessness factor (Figure 2C), resulting in an excellent fit—the loadings of the 2 items on both factors were within an acceptable threshold (>0.3). Removing negatively worded items (items 5 and 8) resulted in an acceptable fit in the absence of any error correlations (model 3, Figure 2D). However, the fit slightly improved when item 7 was also removed in our replication of the Taiwanese version (model 4, Figure 2E).

**TABLE 2:** Goodness-of-Fit of the Confirmatory Factor Analysis Structures of the Control Attitudes Scale-Revised to Classify Omani Patients With Heart Failure (N = 180)

Models		*χ* ^2^	*df*	*P*	CMIN/DF	CFI	TLI	RMSEA	RMSEA 90% CI	SRMR
Model 1 (1 factor)	Crude	60.41	21	.001	2.87	0.850	0.800	0.102	0.073–0.133	0.0782
	Error	49.64	19	.001	2.51	0.891	0.839	0.092	0.060–0.125	0.0664
Model 2 (2 factor)	Crude	18.65	18	.414	1.03	0.998	0.996	0.014	0.000–0.069	0.0471
Model 3 (1 factor)	Items 5 and 8 removed	21.95	10	.015	2.20	0.947	0.921	0.82	0.034–0.128	0.0485
Model 4 (1 factor)	Items 5, 7, and 8 removed	12.16	6	.058	2.03	0.963	0.939	0.076	0.000–0.137	0.0438
Model 5 (1 factor)	Items 5, 6, and 8 removed	8.94	6	.177	1.49	0.982	0.970	0.052	0.000–0.119	0.0379

Abbreviations: CFI, Comparative Fit Index; CI, confidence interval; CMIN/DF, minimum discrepancy divided by degrees of freedom; *df*, degrees of freedom; RMSEA, root mean square error of approximation; SRMR, standardized root mean residual; TLI, Tucker–Lewis Index.

Figure 2)Based on measurement invariance analysis of the former models, we removed item 6 instead of item 7, which resulted in an excellent fit (model 5, Figure 2F).

Multigroup CFA shows variance of models 2 to 4 at the configural level across gender, that is, the global structure does not hold across men and women (eg, item 1 loaded on the helplessness factor among females but not among males [Supplemental Digital Content 1, Model 2, https://links.lww.com/JCN/A380]). While items 1, 5, and 6 were sources of variance of the whole scale across gender, only item 6 was a source of variance at the configural level in all models. Altering this item (model 5) achieved scale invariance at all levels across gender and employment—ΔCFI approached the critical threshold, but other fit indices remained within the acceptable range (Tables 3 and 4).

**TABLE 3:** Invariance of Structures of the Control Attitudes Scale-Revised and Its Shortened Versions (Models 2, 3, 4, and 5) Across Groups of Gender Among Omani Patients With Heart Failure (N = 180)

Models	Invariance Levels	*χ* ^2^	*df*	*P*	Δ*χ*^2^	Δ*df*	*P* (Δ*χ*^2^)	CFI	ΔCFI	TLI	ΔTLI	RMSEA	ΔRMSEA	SRMR
Model 2	Configural	42.70	40	.356				0.991		0.987		0.019		0.0591
	Metric	74.69	48	.008	32.00	8	.001	0.906	**0.085**	0.890	**0.097**	0.056	**−0.035**	0.0744
	Scalar	91.04	50	.000	16.35	2	.001	0.856	**0.050**	0.838	**0.052**	0.068	−0.012	0.0959
	Strict	93.38	54	.001	2.34	4	.674	0.861	−0.005	0.856	0.018	0.064	0.004	0.0957
Model 3	Configural	46.653	24	.004				0.910		0.888		0.073		0.0527
	Metric	78.268	28	.001	31.62	4	.001	0.801	**0.109**	0.787	**0.101**	0.100	**0.027**	0.0644
	Scalar	86.179	29	.001	7.91	1	.005	0.774	**0.027**	0.766	**0.021**	0.105	−0.005	0.1056
	Strict	86.674	31	.001	0.50	2	.781	0.780	−0.006	0.787	−0.021	0.100	0.005	0.1005
Model 4	Configural	32.90	16	.008				0.912		0.890		0.077		0.0511
	Metric	63.17	19	.001	30.27	3	.001	0.770	**0.142**	0.758	**0.132**	0.114	**−0.036**	0.0849
	Scalar	66.82	20	.001	3.65	1	.056	0.756	0.014	0.756	0.002	0.115	−0.001	0.1285
	Strict	68.54	21	.001	1.72	2	.190	0.753	0.003	0.764	−0.008	0.113	0.002	0.1031
Model 5	Configural	28.75	16	.026				0.928		0.909		0.067		0.0433
	Metric	35.14	19	.013	6.39	3	.094	0.908	**0.020**	0.903	0.006	0.069	−0.001	0.0518
	Scalar	35.97	20	.016	0.83	1	.362	0.909	−0.001	0.909	−0.006	0.067	0.002	0.0591
	Strict	35.99	21	.022	0.02	1	.878	0.915	−0.006	0.919	−0.010	0.063	0.004	0.0600

Values in boldface indicate variance.

Abbreviations: CFI, Comparative Fit Index; *df*, degrees of freedom; RMSEA, root mean square error of approximation; SRMR, standardized root mean residual; TLI, Tucker–Lewis Index.

**TABLE 4:** Invariance of Structures of the Control Attitudes Scale-Revised and Its Shortened Versions (Models 2, 3, 4, and 5) Across Groups of Marital Status Among Omani Patients With Heart Failure (N = 180)

Groups	Invariance Levels	*χ* ^2^	*df*	*P*	Δ*χ*^2^	Δ*df*	*P* (Δ*χ*^2^)	CFI	ΔCFI	TLI	ΔTLI	RMSEA	ΔRMSEA	SRMR
Model 2	Configural	39.15	40	.508	—	—	—	1.000	—	1.005	—	0.000	—	0.0472
	Metric	52.55	48	.302	13.20	8	.099	0.983	**0.017**	0.980	**0.025**	0.023	**−0.023**	0.0571
	Scalar	53.45	50	.343	0.90	2	.637	0.987	−0.004	0.985	−0.005	0.020	0.003	0.0569
	Strict	60.44	54	.255	6.99	4	.137	0.976	0.011	0.975	0.010	0.026	−0.006	0.0586
Model 3	Configural	29.96	24	.186	—	—	—	0.974	—	0.967	—	0.037	—	0.0521
	Metric	38.47	28	.090	8.51	4	.075	0.953	**0.021**	0.950	0.017	0.046	−0.011	0.0597
	Scalar	38.57	29	.110	0.10	1	.752	0.957	−0.004	0.956	−0.006	0.043	0.003	0.0592
	Strict	38.89	31	.156	0.32	2	.581	0.965	0.012	0.966	0.010	0.038	0.005	0.0596
Model 4	Configural	13.38	16	.645	6.39		—	1.000	—	1.020	—	0.000	—	0.0422
	Metric	19.77	19	.408	0.001	3	.094	0.995	0.005	0.995	**0.025**	0.015	**−0.015**	0.0524
	Scalar	19.77	20	.472	0.014	1	.976	1.000	−0.005	1.001	−0.006	0.000	0.000	0.0522
	Strict	19.79	21	.535	—	1	.905	1.000	0.000	1.007	−0.006	0.000	0.000	0.0525
Model 5	Configural	12.86	16	.683	—	—	—	1.000	—	1.024	—	0.000	—	0.0377
	Metric	18.45	19	.493	5.59	3	.133	1.000	0.000	1.004	**0.020**	0.000	0.000	0.0479
	Scalar	19.08	20	.517	0.63	1	.429	1.000	0.000	1.006	−0.002	0.000	0.000	0.0475
	Strict	19.30	21	.566	0.22	1	.637	1.000	0.000	1.010	−0.004	0.000	0.000	0.0471

Values in boldface indicate variance.

Abbreviations: CFI, Comparative Fit Index; *df*, degrees of freedom; RMSEA, root mean square error of approximation; SRMR, standardized root mean residual; TLI, Tucker–Lewis Index.

As shown in Table 5, the CAS-R strongly correlated with all the shortened versions, in support of the convergent validity of the shortened forms. All CAS-R measures had significant negative correlations with the PHQ-2 (criterion validity). While the helplessness subscale had a positive association with depression, such an association was not significant. The parent scale, CAS-R, had no correlation with helplessness, indicating poor convergent validity of this subscale. On the other hand, the 3 shortened forms had strong negative correlations with it—our CAS-R 5 expressed the strongest among all.

**TABLE 5:** Convergent and Criterion Validity of the Control Attitudes Scale-Revised Among Omani Patients With Heart Failure (N = 180)

Variables	1	2	3	4	5
1. CAS-R	—				
2. Control/CAS-R6	.971^a^	—			
3. Helplessness	.005	−.236^a^	—		
4. CAS-R 5 Taiwanese	.961^a^	. 993^a^	−.252^a^	—	
5. CAS-R 5	.953^a^	.998^a^	−.315^a^	.993^a^	—
6. PHQ-2	−.257^a^	−.275^a^	.106	−.264^a^	−.268^a^

Abbreviations: CAS-R, Control Attitudes Scale-Revised; PHQ-2, Patient Health Questionnaire-2.

a Correlations are significant at values below .01 and .05, respectively.

As for known-group validity, Welch *t* test revealed significantly low levels of the CAS-R and all its shortened forms in the depressed group relative to the nondepressed group (all *P* values <.05, see Supplemental Digital Content 2, https://links.lww.com/JCN/A381). Helplessness scores were higher in the depressed group, but the difference in this variable between groups was marginally significant *t*(135.3) =1.94, *P* = .055.

As for internal consistency, the results show low reliability of the CAS-R. Among all items, items 5 and 8 expressed the lowest values of item total correlations (Table 1). The removal of both items considerably improved scale reliability (0.68 vs. 0.56). Nonetheless, it was necessary to remove another item, which was involved in scale variance despite its high values of loading and item total correlation, resulting in a slight reduction in scale reliability, which was originally below acceptable levels.

## Discussion

The results confirm that the Arabic CAS-R is not unidimensional. Its structure is potentially undermined by a method effect arising from the inclusion of negatively worded items (5 and 8). Discarding those items remarkably improved the fit of the scale, its measurement stability, and reliability. However, item 6 persistently contributed to scale variance across gender and marital groups despite its high factor loading (>0.5). Altering this item produced a 5-item shortened form (CAS-R 5) with exceptional fit and measurement invariance across groups.

Negative items are frequently included in psychometric scales as a way to counteract acquiescence bias by forcing respondents not to automatically agree/disagree with some items. However, empirical evidence confirms that this approach produces considerable alterations in scale construct validity and reliability. On the contrary, changing those items into their positively worded equivalents results in significant systematic improvements in scale reliability and factor structure.^26^ In accordance, EFA and CFA indicate that negatively worded items (5 and 8) on the CAS-R did not only cause a considerable reduction in scale reliability but also altered its structure by correlating with each other and with other items (item 3). Their impact was obviously demonstrated through the appearance of a minor negative factor (helplessness; model 2: Figure 2C). This factor was weak in terms of variance explained (37.85% in model 2 vs. 46.94% in model 3), criterion validity (correlation with the PHQ-2 *r* = 0.106), convergent validity (item total correlations: *r* = 0.22 and correlation with the CAS-R *r* = 0.005), and scale reliability (*α* = 0.33). Additionally, 2 positively worded items (1 and 2) had significant negative cross-loadings on both factors, and their loadings were even higher than that of item 5 (*λ* = 0.28). Yet, including those items in reliability testing resulted in a drastic reduction in its originally low internal consistency (*α* = 0.24). While item 6 persistently contributed to invariance of all versions of the CAS-R (Supplemental Digital Content 1, Models 2–4, https://links.lww.com/JCN/A380), items 1 “If I do all the right things, I can successfully manage my heart condition” and 5 “No matter what I do, or how hard I try, I just can’t seem to get relief from my symptoms” on the helplessness factor were associated with scale variance across men and women (Supplemental Digital Content 1, Model 2, https://links.lww.com/JCN/A380). The contribution of item 1 to scale variance disappeared following the removal of items 5 and 8, but item 6 was still a source of variance. Altering this item resulted in a cleaner factor structure with optimal fit (Figures 1D and 2F) and absolute measurement invariance (Tables 3 and 4).

Our findings are consistent with those of Moser et al^4^ who ignored the second factor in the first testing of the CAS-R, albeit the percentages of variance explained when 2 or 3 items were removed are evidently greater than those produced in Moser’s study (46.94%, 49.03%, and 48.93% vs. 34%, 36%, and 39%), suggesting that the removed items have little contribution to the underlying latent construct.^4^ They also coincide with similar studies, which globally evaluated the scale through simple exploratory methods.^11,12^ Correspondingly, they are in agreement with the results of studies using advanced measures to evaluate the performance of the scale on the item level. For example, evaluations of the Chinese version among Taiwanese patients through EFA^5^ and CFA^3^ revealed exceptionally poor loadings of items 5, 7, and 8. Discarding the 3 items resulted in a well-fitting 5-item version (model 4), which expressed higher internal consistency and stronger associations with measures of self-care, social support, perceived stress, and depression in analyses adjusted for age, gender, years of education, and marital status, among others.^3^ In line, item-response theory analysis shows that the CAS-R is a good predictor of lower-moderate scorers but not high scorers, with many items liable to elimination because of their poor discrimination ability, which manifested with flat item information curves, for example, items 3, 4, 5, and 8. Items 2 and 6 displayed bimodal distributions on the item information functions. The authors suggested altering response categories to have binary (yes/no) or 3 categories instead of 5.^1^

Having a look at the items that expressed error correlations with items 5 or 8 (eg, item 3) and items that cross-loaded on helplessness (items 1 and 2) or produced variance in the scale (eg, item 6), the wording of most of those items seems to introduce irrelevant attributes to the underlying construct “perceived control of one’s cardiac disease and its symptoms.” For example, “right things” is a vague phrase in item 1 “If I do all the right things, I can successfully manage my heart condition.” Is it about self-care, personal relationships, lifestyle, …? The same applies to item 3 “When I manage my personal life well, my heart condition does not bother me as much.” The presence of redundant and construct-irrelevant items on a measure threatens the meaningfulness and accuracy of its scores, making inferences drawn from them as incorrect, ending with impractical decisions.^16^ Although, we and the Taiwanese authors^3,5^ have dropped some items, removing items 1 and 2 from reliability testing of the control dimension/CAS-R 6 did not cause a reduction in its reliability (data not shown). Accordingly, the remaining set of items still needs further examination for the wording to ensure that it strictly targets perceived control of the cardiac condition rather than related but irrelevant aspects such as “right things” or “personal life.”

Although many studies report high scale reliability (Cronbach’s *α* = 0.87,^2^ 0.94,^12^ 0.88,^1^ and 0.89^5^), including the former evaluation of the Arabic CAS-R in Jordan (*α* = 0.85),^10^ the reliability of our CAS-R was low (Table 1). This finding is consistent with reports of the Portuguese and the Taiwanese/Mandarin versions (*α* = 0.65 and 0.66, respectively).^5,11^ As shown in Table 1, removing items 5 and 8 moved scale reliability from poor to acceptable in all shortened versions (Table 1). As in our study, the Mandarin CAS-R 5 (items 5, 7, and 8 were removed) expressed higher reliability than the full scale (*α* = 0.79 vs. 0.66).^5^ This indicates that items removed do not contribute to the homogeneity of the scale, particularly items 5 and 8 had the lowest item total correlations (Table 1). This result is consistent with those of EFA and CFA, which show low loadings of both items on the main factor. However, the reliability of all the shortened versions was at the lower bound of acceptable range after the removal of those items; item total correlations were acceptable but not high, which coincides with the idea of revising the wording of the items to avoid confusion. Low scale reliability in the present sample may pertain to age-related normal cognitive decline (patients’ age was mostly advanced), level of education (patients were largely illiterate), and culture influence (Oman is a small country with its unique cultural background).

As for the criterion validity of the CAS-R, the whole scale and the shortened versions negatively correlated with the PHQ-2 at the same level of significance (all *P* values <.01, Table 5). In known-group validity testing, we captured significantly lower scores of all the CAS-R versions in depressed patients relative to the nondepressed participants. This result indicates that higher patients’ perceived control of their heart failure condition may be protective against psycho-pathogenicity as indicated by tests of between-group differences. This finding is in line with studies reporting a negative association between perceived control and depression in this population.^3,5,6,8,11^ However, the cross-sectional nature of the data disqualified testing the direction of this relation, a limitation that future studies are recommended to avoid through the use of longitudinal data.

### Strengths and Limitations

Both EFA and CFA were used in this study to evaluate various structures of the CAS-R. An extra merit of the present study is evaluating the measurement invariance of the CAS-R for the first time and providing remedial action. Producing a clean factor structure that performs evenly across various groups (eg, men and women) can support meaningful inferences of patients’ perceptions of control to guide the development and evaluation of specific nursing interventions.^16^ Although suitable for conducting the tests, the sample was relatively small as Omani populations are limited in number, making the recruitment of a large sample of patients with heart failure not an easy task. Criterion validity was limited to the use of depression only, while the reliability of the CAS-R and the measure of depression (PHQ-2) was suboptimal in the present sample, despite extensive reports on their acceptable reliability, especially of the latter in various populations, including Saudi mothers of children with disabilities.^18^ However, slight variations in scale reliability have been noticed with a reported impact of cultural differences. In this respect, the reliability of the PHQ-2 in a former study involving Spanish-speaking immigrants in Chile was only acceptable (*α* > 0.7) for Peruvian and some other Latin American subsamples but not for Colombian subjects.^27^ Therefore, variations in the reliability of the PHQ-2 across our Omani patients (*α* = 0.51) and Saudi mothers (*α* = 0.76) may be attributed to cultural differences and the influence of sample characteristics (our respondents were sufferers of a specific health condition who were predominantly illiterate older adults); the method of scale delivery in the current study was also different involving interviews on the phone or in person. Therefore, the results of criterion validity testing may be interpreted with caution. Moreover, there is no guarantee that data collection approaches (phone and face-to-face interviews) operated similarly in the present study. Nonetheless, results of a study comparing both methods show identical similarity in the full set of data obtained through both interviewing approaches, albeit face-to-face interviews were longer with more wording.^28^ The cross-sectional design limits some necessary tests, such as test–retest reliability and the investigation of causal relations between perceived control and depression. The sample comes from one Arab country in the Gulf region, and the results may not be generalizable to other Arab contexts.

## Conclusions

Exploratory factor analysis and CFA show poor fit of the unidimensional and bidimensional CAS-R, along with error correlations and cross-loadings on a weak negative factor of helplessness. Eliminating negatively worded items produced a well-fitting unidimensional version, but it was variant across gender and marital status, a problem that was addressed by altering item 6 (CAS-R 5). The reliability of the CAS-R 5 was higher than that of the original scale, and it demonstrated adequate convergent, criterion, and known-group validity through correlations with the CAS-R and PHQ-2 as well as by displaying low levels of control among depressed respondents. The scale would benefit from further revision of its wording since some items may be to some extent irrelevant to perceived control of heart failure, for example, inquiring about “personal life” and “right things” in items 1 and 3. Validating the scale in other Arab countries using a longitudinal design, with a focus on measurement invariance, is warranted.

## Supplementary Material
