# Supplementary material for: The Arabic Control Attitudes Scale-Revised: Method Effect (Negatively Worded Items) and Measurement Invariance as Threats to Its Construct Validity Are Remedied in a 5-Item Version With Improved Performance
Source: J Cardiovasc Nurs. 2026 Jan 28;41(5):E370–8. doi: 10.1097/JCN.0000000000001294 (PMC13456572; doi:10.1097/JCN.0000000000001294)
Supplement: Supplementary file 1 [file jcn-41-e370-s001.pdf]

Gender

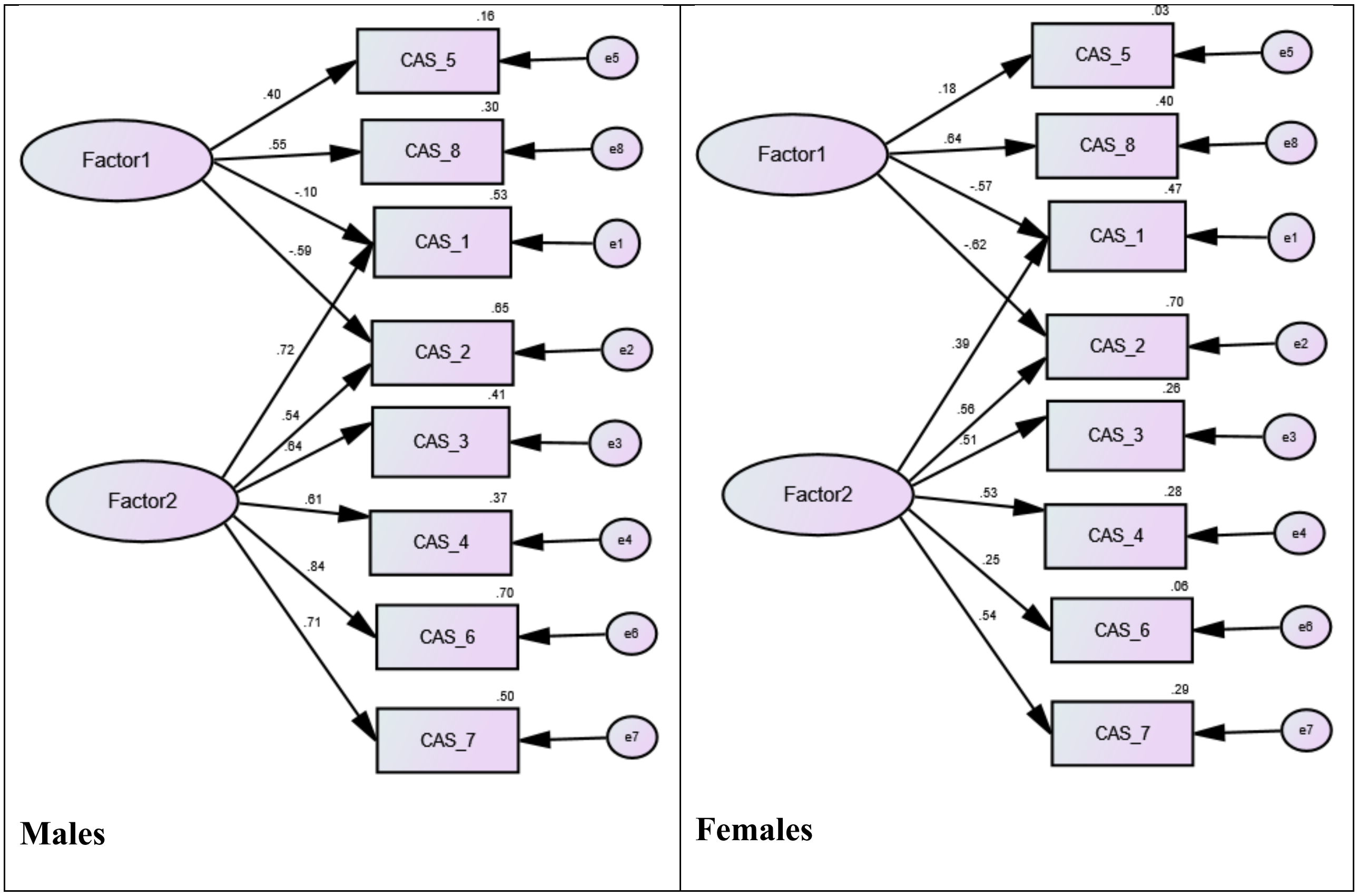

Model 2

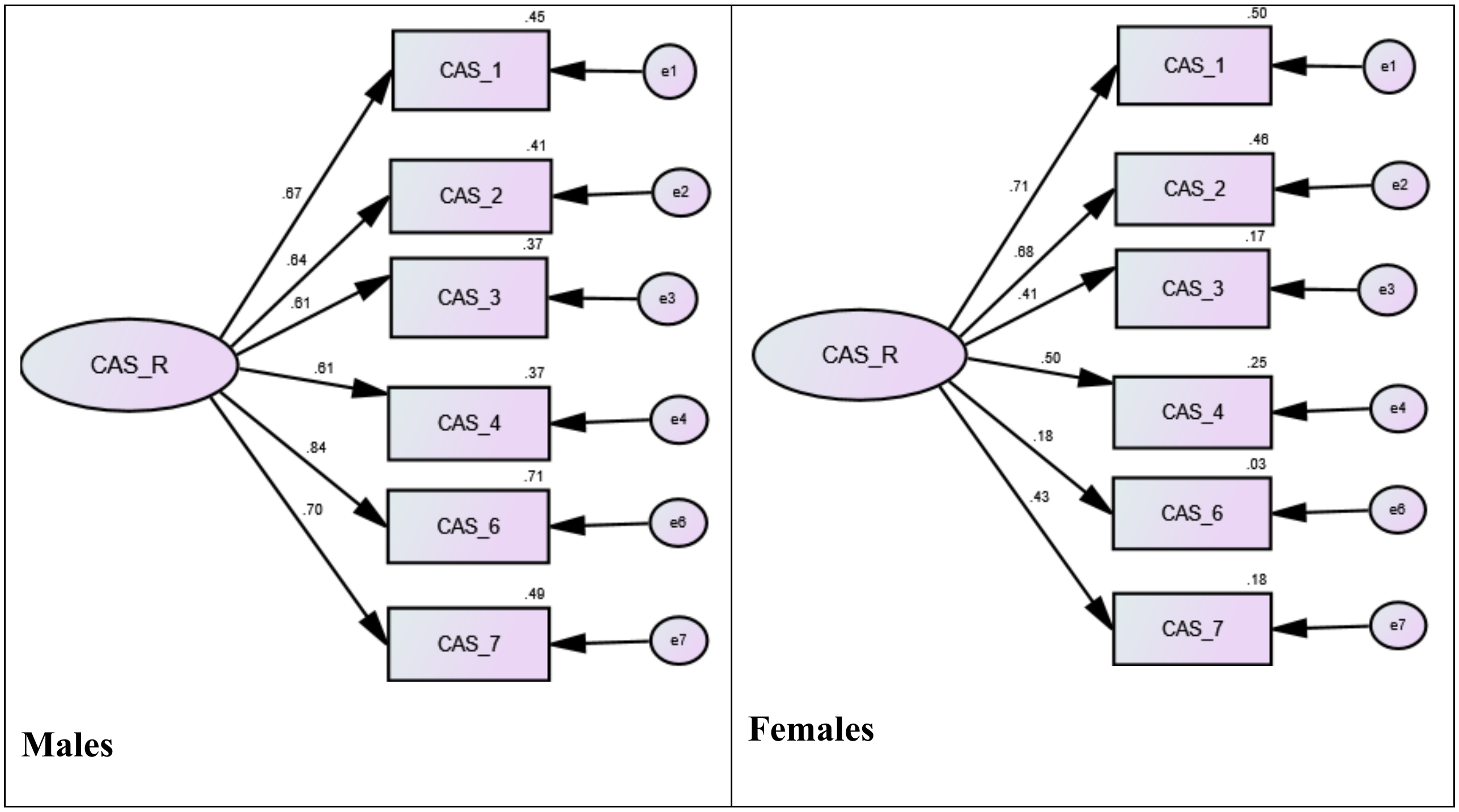

Model 3

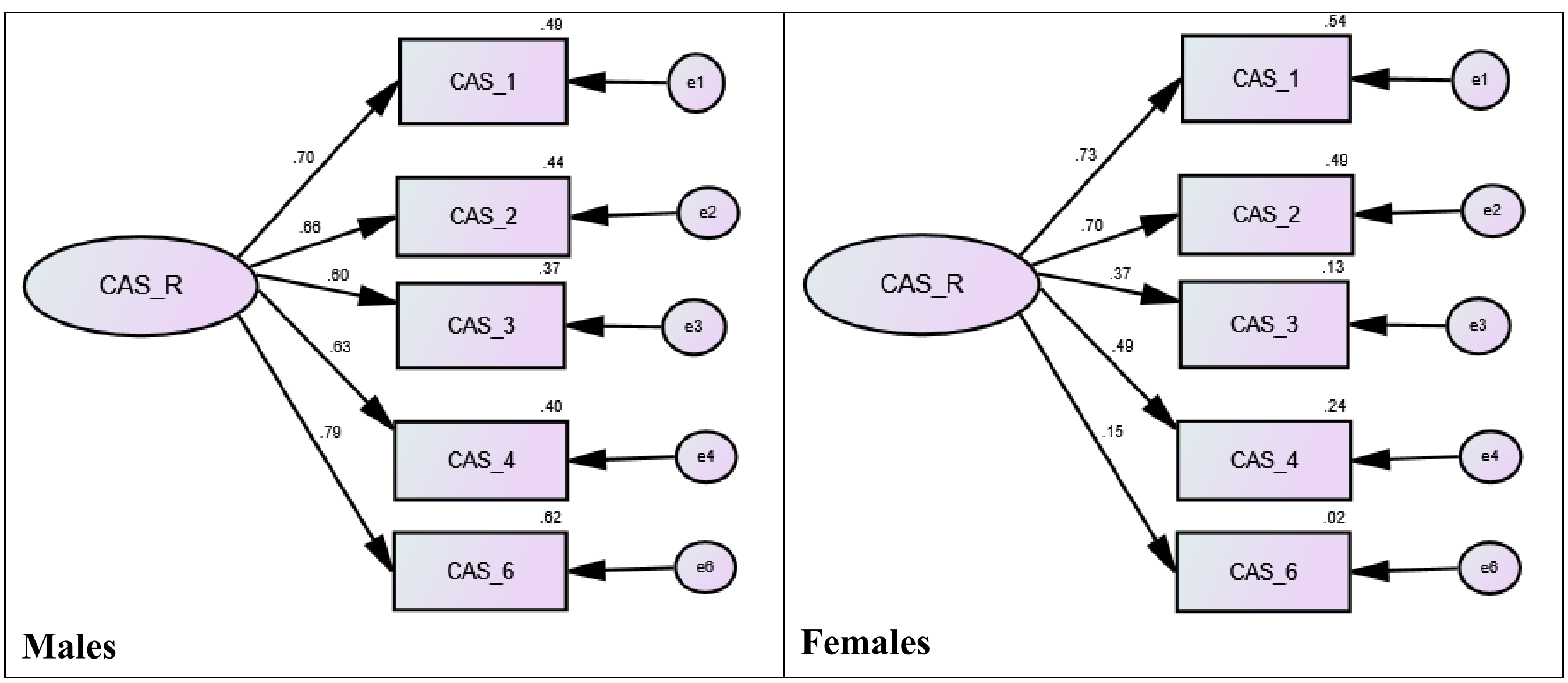

Model 4
